# Physiognomy

**Published:** 1919-07-12

**Authors:** 


*To the Editor of* THE HOSPITAL.

### Physiognomy.

*To the Editor of* THE HOSPITAL.

SIR,—The article by "Caduceus," which appeared on June 21, has only just come into my hands, but I should like to confirm the writer's contention that "there are complete, thorough-going resemblances to be found" by two instances, one of which any man can test. A few years before the war the most notable ornament of the Hotel Dom at Cologne was the head waiter, who in everything but his considerable height was a living replica of Napoleon. What was his name, I do not know; but his face was so striking that I ventured to bridge our formal relations, while he was uncorking an excellent bottle of Trettenheimer, to ask if he was not called Bonaparte by his friends. He replied that the resemblance had become a nuisance, because it allowed him to pass nowhere without comment. The case is interesting, since it will (I hope) suggest to "Caduceus" the question how far physiognomy is an indication of ability or character. Luck plays a greater part than we like to admit in life, and it requires brains to be a good head waiter at a big hotel in Cologne. But, even so, should one not expect a man with Napoleon's physiognomy, who was also past his youth, to rise beyond a head-waiter's position? Perhaps, however, he won the Iron Cross in the war. If not, what inference are we to draw from a striking but sterile physiognomy?

The second instance is the resemblance borne by Mr. W. B. Yeats to a well-known bookseller at Cambridge whose name I omit only because he, too, is "burdened with an honour" unto which he was certainly born. The bookseller, however, is not a poet but a publisher; for I presume that a poet who was a publisher would have less hesitation than most men to publish verse under his own name. With these instances to set beside his own and Samuel Butler's, I invite "Caduceus" to write a further article on physiognomy, in which its value as an index to character and ability shall be, if not determined, yet described. It is odd that Butler's notes do not deal with the point.

Yours faithfully,

OMEGA.

